# Predictive models for perceived convenience of accessing outdoor activities among elderly with physical disabilities in rural China

**DOI:** 10.1186/s12889-024-18311-5

**Published:** 2024-03-12

**Authors:** Qi Xu, Youyi Lin, Yiqi He, Xianhong Zhou, Jinhai Liu, Dewang Shen, Fan Wu, Xin Lin, Yun Zhang, Taibiao Li, Tiebin Yan

**Affiliations:** 1 The Fifth Hospital of Xiamen, 361101 Xiamen, China; 2 Macau University of Science and Technology, Macao, China; 3 Department of Rehabilitation Medicine, Sun Yat-sen Memorial Hospital, Sun Yat-sen University, 510120 Guangzhou, China; 4 The Engineering Technology Research Center of Rehabilitation and Elderly Care of Guangdong Province, 510120 Guangzhou, China; 5 Renowned Physician Tiebin Yan Office, XiaMen, China

**Keywords:** Quality of life, Access outdoor activities, Gender, Elderly, Physical disability, Structural equation models

## Abstract

**Background:**

The elderly, especially those with physical disabilities, often encounter barriers that prevent them from accessing outdoor activities. Their perceptions of the convenience of accessing outdoor activities may be influenced by various factors including their health, the social context, and/or planned behavior. This study aimed to develop predictive models that identify the principal determinants of perceived convenience among this demographic, and it also examined the disparities observed between genders.

**Methods:**

This was a cross-sectional survey of 1216 community-dwelling older people with physical disabilities in rural China. Grounded on the rehabilitation concepts and the theory of planned behavior, structural equation models integrated health and social behavior factors were constructed to predict perceived convenience of accessing outdoor activities. The standardized coefficients explained the contributions of various factors to the variance.

**Results:**

The final structural models demonstrated good fit for both female and male participants. Perceptions of the convenience of accessing outdoor activities among both women and men were directly impacted by their physical functioning and their intention to participate, and indirectly by medical expenditure, subjective norms, pain, and role limitation in emotional interactions. Positive mental health was more influential for women, while men were more influenced by subjective norms.

**Conclusions:**

Structural equation models have effectively predicted the self-reported convenience of accessing outdoor activities, underscoring the importance of functional and behavioral rehabilitation. Furthermore, gender-sensitive rehabilitation programs are advised to promote engagement in outdoor activities among elderly individuals with physical disabilities.

## Introduction

Outdoor activity has important physical and mental health benefits for the elderly. Liu has described an outdoor physical activity intervention for community-dwelling older adults in Hong Kong using public outdoor exercise facilities. He found that it significantly increased physical activity and that the elevated physical activity tended to be maintained over the following six months [1]. Outdoor activity such as going shopping is also useful for restoring and maintaining elderly persons’ independence [2].

These benefits make it important to understand what controllable factors best improve the convenience of accessing outdoor activities (CAOA) for the elderly, especially those with physical disabilities who may face barriers impeding such access. Only a few studies have evaluated the determinants of CAOA for the elderly in rehabilitation healthcare context. Swedish researchers have observed walking, the most common outdoor recreational activity among the community-dwelling elderly, and observed that disability and long-term illness were common [3]. More than half of those they observed had disengaged from walking which they had practiced in the previous year [3]. They cited declining health as the most common reason, but some found it too demanding or mentioned a change in their social context [3]. But in another study elderly persons continuing active social activity outdoors reported happiness, and that positive feelings made outdoor activity appealing and increased their likelihood of persisting [4]. A recent scoping review has identified factors that facilitate participation in adaptive outdoor physical activity for people with physical disabilities. It categorized the factors as either: intra-personal, social-environmental, physical-environmental, or policy-related [5]. The classification was based on a social ecology primarily focusing on social and environmental factors [5].

In the midst of complex influences, this study aims to pinpoint determinants in rehabilitation healthcare. It is grounded in the Global Stakeholder Initiative’s definition of rehabilitation interventions by Cochrane Rehabilitation, which focuses on enhancing individual capacity through body structures, functions (physical and psychological), activities, participation, and contextual performance factors like the physical, social, and attitudinal environment. Our hypothesis, based on this framework, is that physical and mental function, along with social factors, are key predictors for CAOA [6].

There has been a lack of research evaluating access to outdoor activities for older people with physical disabilities (OPWPD). And there has been limited analysis using statistical modeling to demonstrate the factors directly and indirectly predicting CAOA. This research therefore applied structural equation modeling (SEM), a comprehensive statistical approach useful for analyzing intricate relationships between directly observed and indirectly observed (latent) variables [7]. It concurrently addresses systems of multiple linear equations and incorporates techniques such as regression analysis, factor analysis, path analysis, and the modeling of latent growth curves [7].

Since deteriorating health functioning can influence accessing outdoor activity [3, 4], this study was designed to test for any relationship between the CAOA and health-related quality of life, including physical functioning (Hypothesis H1-1) and mental health (H1-2, H1-3). Social factors such as medical expenditure could also predict CAOA. The China Health and Retirement Longitudinal Study (CHARLS) of elderly Chinese persons covering 2011 to 2015 revealed that the higher medical expenses associated with a greater acceptance of diagnosis and treatment usefully signal declining health [8]. Carlson and his colleagues found that in the United States 11.1% of total health expenditure was associated with adults’ physical inactivity [9]. In the United States annual per capita health care expenditures for persons with a disability increased from $13,395 in 2003 to $17,431 in 2015. In contrast, annual spending for persons without a disability remained relatively stable (at about $6700) [10]. So the CAOA for those with a disability has economic implications. Thus this study evaluated the status of its subjects monthly and their annual healthcare costs to test the hypothesis that medical expenditure could predict the CAOA directly (Hypothesis H1-4) or indirectly predict it through their impaired physical functioning (H3-1).

Social factors such as subjective norms help to determine an individual’s perception of social pressure or friendly support to engage in or refrain from certain behavior [11]. Subjective norms are thus part of one’s social context [12] which influences one’s behavior [13], including one’s physical activity [14]. Subjective norms in societies worldwide often encourage negative attitudes towards persons with a disability [15]. A review found that while many studies have identified social support and attitudes as barriers to adaptive outdoor physical activity for the people with a physical disability, only one study proposed solutions to reduce stigma and enhance participation [5]. Subjective norms were therefore also considered in this study as potential predictors of the CAOA for those with a disability. The hypothesis was that subjective norms directly influence the CAOA (H1-5) or indirectly influence it by impacting the physical activity of OPWPD in particular (H3-2). The questions, “Do you think society respects people with disabilities today?” and “Do people have positive attitudes towards you?” were used to quantify subjective norms.

Pain may be a trigger for inability to fulfill one’s functional (activity) expectations. It causes both physical and psycho-social dysfunction. This is termed the fluid concept of pain disability [16]. This study hypothesized that bodily pain that affects physical functioning (H3-3) may also influence convenience perceptions (H1-7). The study’s body pain indicators came from domains of the short form 36-item health survey (SF-36) instrument [17]. There were two variants: body pain experienced in the previous one month, and the extent to which pain interfered with normal activities.

**Fig. 1:**
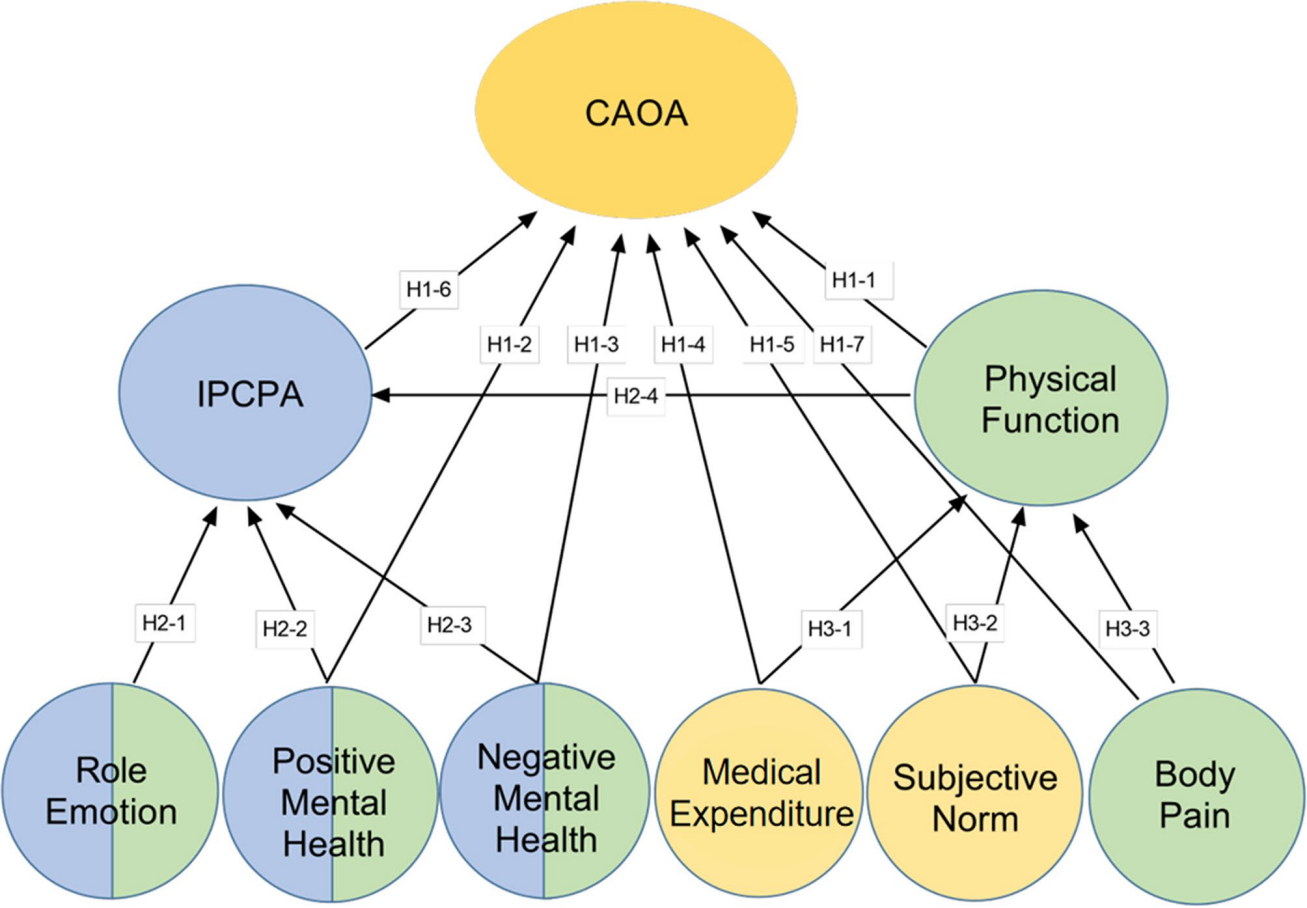
Hypothesized integrated SF-36 and social-behavioral model predicting CAOA. *Notes* SF-36 = short form 36-item health survey, CAOA = convenience of accessing outdoor activities, IPCPA = intention to participate in community physical activities, H = hypothesis. Quality of life, social factors and behavioral factors are highlighted in green, yellow and blue respectively. The latent factors in blue come from the SF-36 instrument

A partial path model for predicting CAOA was thus tested. Please refer to Fig. 1. Quality of life factors are highlighted in green in the figure and social factors are highlighted in orange.

**Fig. 2:**
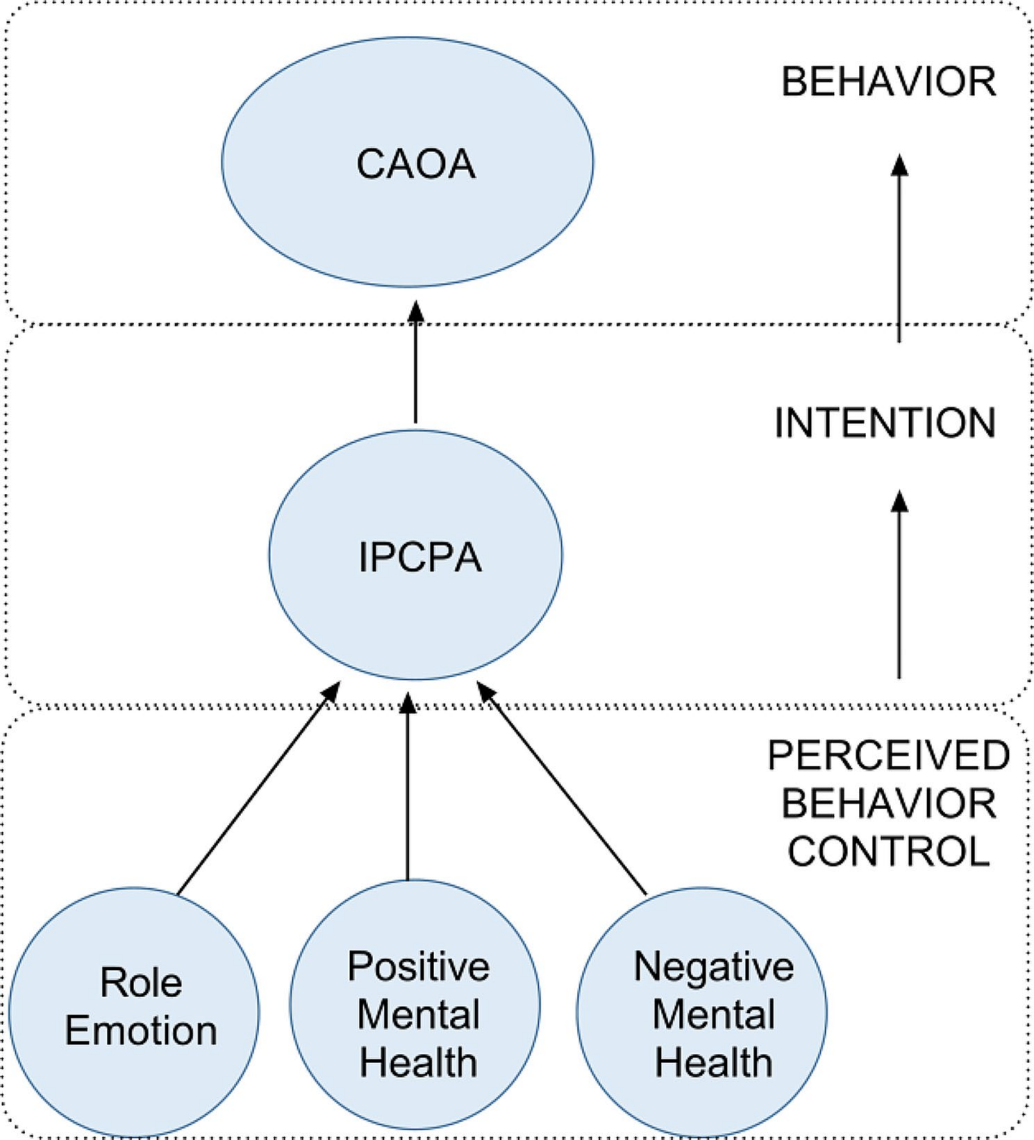
Behavioral factors in the hypothesized model based on the theory of planned behavior

A review by Derakhshan and his colleagues reports that while outdoor activities are typically viewed as planned behavior, research reports rarely mention employing any behavioral theories to promote adapted outdoor physical activity among people with impaired mobility [5]. This despite the fact that there is evidence supporting its effectiveness [5]. In response, this study used a theory of behavior as a foundation in formulating some of its hypotheses. The theory of planned behavior is a widely-used basis for predicting behavior and behavioral intentions [18]. The theory suggests that behavior arises from behavioral intentions, which are determined by behavior control perceptions. Those are a belief in one’s ability to complete something [19]. This logic is illustrated in Fig. 2. Based on the theory of planned behavior, this study used the intention of participate in community physical activities (IPCPA) to represent outdoor activity intentions. Scores on the role emotion domain of the SF-36 instrument (role limitation in emotional interactions) represented behavior control perceptions with respect to outdoor activities. The role emotion domain reflects three emotional effects: reducing time spent, accomplishing less, and not being careful. This study tested the idea that self-perceived CAOA could be directly affected by IPCPA (H1-6), which could be predicted from role emotion scores (H2-1). Calogiuri has extended the theory of planned behavior by adding positive and negative psychological states as aspects of perceived behavioral control to study outdoor activity behavior. As might be expected, greater enjoyment, more positive emotions and greater stress relief were found to predict stronger intentions to pursue outdoor physical activity in the future [20]. The hypothesis was that either a positive (H2-2) or a negative (H2-3) state of mental health could influence IPCPA. Clinical experience certainly suggests that one’s level of physical functioning should affect IPCPA (H2-4). The behavior factors (intention and perceived behavior control) are highlighted in blue in the predicted model. Because perceived behavior control factors also belong to SF-36’s health-related quality of life component, therefore they are highlighted half in blue and half in green in Fig. 1.

Rigby’s group reports [21] a review which highlighted that researchers and practitioners must recognize gender differences that influence opportunities for health-enhancing outdoor walking. The study revealed participation biases based on gender, with men and women showing distinct preferences [21]. The men predominantly joined walking programs to share experiences with other men in the absence of female company, although this trend may decrease with age as men start valuing strong female role models [21]. While women preferred the social connection and positive feelings associated with mixed walking groups [21]. Besides, persons with a disability too may exhibit gender differences in the basic and instrumental activities of daily living [22]. Research has shown that gender can influence the experience and expression of pain, and also coping mechanisms [23]. Any such gender differences in the social context, mental health, physical function, body pain and other pathways influencing CAOA perceptions would be of interest. This study therefore specified gender models to explore any gender differences.

## Methods

### Participants

This was a cross-sectional study surveying a current situation. The investigation was conducted between May and December of 2019. A total of 211 people were involved in conducting it: 27 medical workers from Xiamen’s Fifth Hospital, 118 liaison officers for the disabled and 66 volunteers from villages and towns in Xiamen’s Xiang’an District. All of the investigators received preparatory training before administering the questionnaire survey in all of the villages and towns of Xiang’an District.

**Fig. 3:**
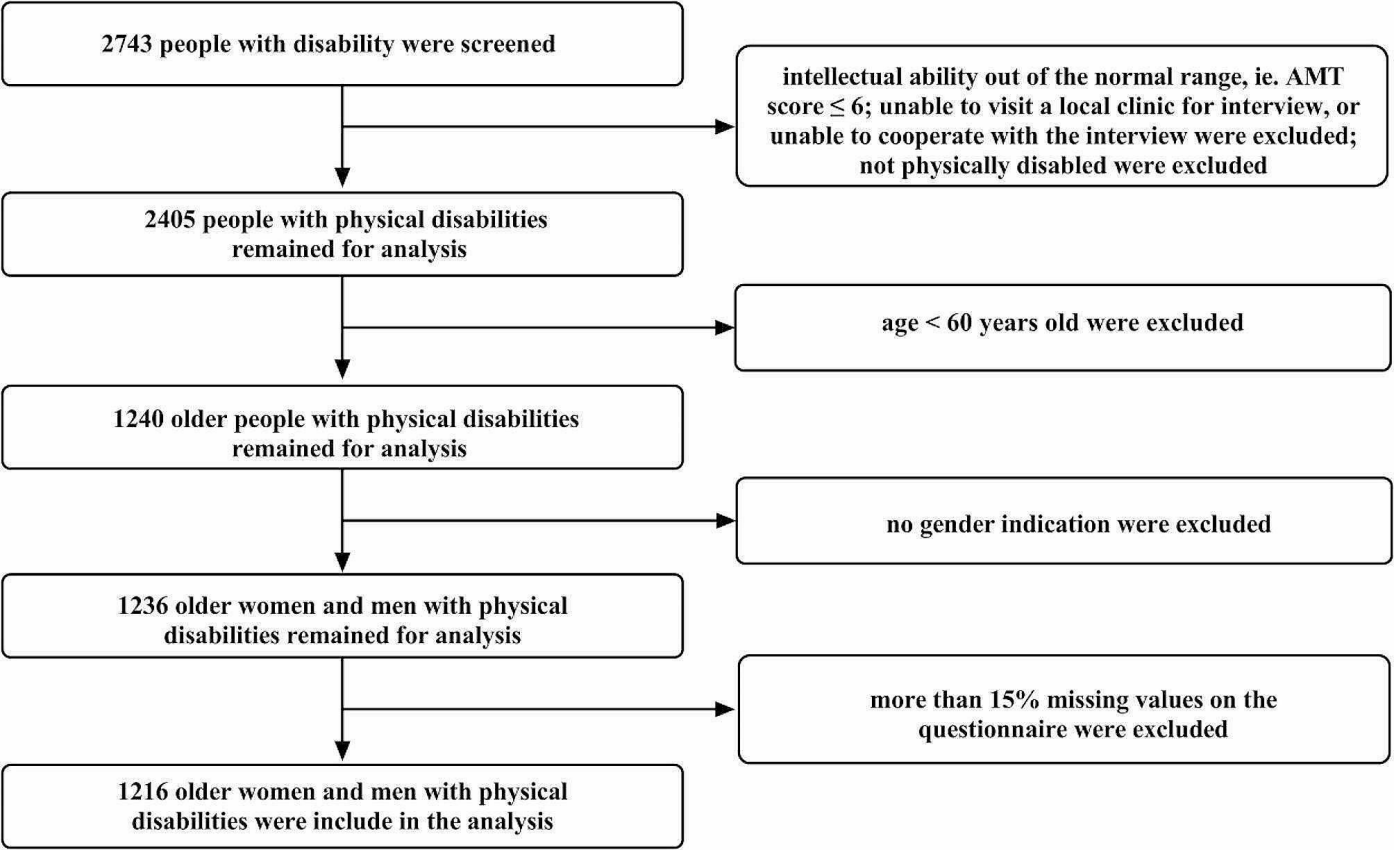
Inclusion and exclusion flowchart. *Note* AMT = Abbreviated Mental Test

In 2019, the district had an estimated population of 376,100. Initially, all 2,743 residents registered as disabled with the China Disabled Persons’ Federation were regarded as potential candidates for inclusion. The further inclusion criteria were that the subject was physically disabled [24]; they were able to visit a local clinic for the interview and willing to cooperate; an Abbreviated Mental Test score (Chinese version) > 6) [22]; age 60 or older. Those who failed to indicate their gender or had more than 15% of the responses missing were excluded. Refer to Fig. 3 for the criteria used in determining who was included or excluded. That produced a group of 1216 elderly persons with a disability for investigation, of whom 51.2% were women. Their demographics are described in Table 1.

**Table 1:** Demographic characteristics of the participants

Variable	Female N = 623 (51.2%)		Male N = 593 (48.8%)	
	N ( %)	Missing Records (N)	N ( %)	Missing Records (N)
Age, years		0		0
≤ 69	213 (34.2%)		327 (55.1%)	
70 − 79	180 (28.9%)		177 (29.8%)	
80–89	181 (29.1%)		77 (13.0%)	
90–99	49 (7.9%)		12 (2.0%)	
Education		5		7
None	372 (60.2%)		76 (13.0%)	
Elementary	199 (32.2%)		313 (53.4%)	
Secondary or over	47 (7.6%)		197 (33.6%)	
Marital status		16		9
Married	245 (40.4%)		391 (67.0%)	
Single	3 (0.5)		30 (5.1)	
Widowed	310 (51.1)		79 (13.5)	
Divorced	48 (7.9)		83 (14.2)	
Separated	1 (0.2)		1 (0.2)	
Medical expenditure				
monthly medical spending	≥ ¥2000: 63 (10.3%); ¥1000–1999: 87 (14.3%); <¥1000: 459 (75.4%)	14	≥ ¥2000: 41 (7.1%); ¥1000–1999: 81(13.9%); <¥1000: 459 (79.0%)	12
maximum annual medical spending	≥ 4000: 92 (15.3%); ¥3000–3999: 36 (6.0%); ¥2000–2999: 78 (13.0%); ¥1000–1999: 101 (16.8%); <¥1000: 295 ( 49.0%)	21	≥ 4000: 83 (14.3%); ¥3000–3999: 34 (5.8%); ¥2000–2999: 89 (15.3%); ¥1000–1999: 79 (13.6%); <¥1000: 295 (50.9%)	13

*Note* ¥ = Chinese yuan

The survey had two parts. The first part solicited general information. It was administered in offices of the China Disabled Persons Federation or at the homes of people with disabilities. The second part collected information relevant for rehabilitation. It included the SF-36 instrument, which was filled in by the people with disabilities themselves with guidance from the survey staff. Some could not fill in the questionnaire by themselves, so it was filled in by the survey personnel with face-to-face explanation of the items. All elderly participants scored above a 6 on the Abbreviated Mental Test (Chinese version) score, indicating intact cognitive abilities [22]. However, a small number faced challenges with understanding Mandarin or had hearing impairments. To ensure accurate understanding, familiar caregivers were present to aid communication, relaying questions and responses when necessary. Questions were given in writing, and participants were encouraged to use their usual assistive listening devices. Clear verbal instructions and non-verbal confirmation methods were employed to avoid misunderstandings, thereby maintaining the integrity of the collected data. In order to ensure the quality of the procedures, the lead investigator conducted regular spot checks and provided supervision.

### Instrument scaling

The short form 36-item health questionnaire is a very popular instrument for evaluating health-related quality of life. It has demonstrated very good psychometric properties, with high internal consistency and good test-retest reliability among people of different ages and health states [25–27]. It covers both physical and mental health. Its eight domains are role emotional, role physical, mental health, physical function, body pain, general health, vitality, and social functioning [17]. The Chinese version of the SF-36 is based on American norms [28, 29]. For this study, the original categorical rank of each item was retained, but the instrument’s scaling was reset so that a higher score consistently indicated better functioning or condition.

CAOA was quantified by asking two questions. “Can you easily access the outdoors for physical activities?” with the responses very inconvenient (1), not convenient (2), generally convenient (3), very convenient (4), and absolutely convenient (5) offered. The other question was, “How often do you go out?” for which the choices were less than five times within six months (1), one or two times a month (2), once a week (3), or three times a week or more (4).

Each subject was also asked whether they were willing to engage in physical activities in the community and whether they expected to have a chance to do so. The replies were scored as (1) for no or (2) for yes.

Subjective norms were quantified by asking, “Do people have positive attitudes towards you?” scored as (1) for not good, despise me, (2) for generally and (3) for very good, respect me. The subjects were also asked, “Do you think society respects people with disabilities today very little (1), little (2), generally (3), very much (4), or absolutely (5)?”

Each respondent’s medical expenditure was evaluated by asking them their monthly medical spending and their maximum annual medical spending. Monthly medical spending were scored as > ¥2000 (1), ¥1000–1999 (2), or <¥1000 (3). Maximum annual medical spending were scored as ≥ 4000 (1), ¥3000–3999 (2), ¥2000–2999 (3), ¥1000–1999 (4), or <¥1000 (5).

### Statistical analyses

Exploratory factor analysis was used to verify the number of potential variables and their relationships through applying varimax rotation [30]. The structural equation modeling used version 1.1.0 of the Scientific Platform Serving for Statistics Professionals supplied by Suzhou Zhongyan Network Technology [31]. The graphical representation of the structural equation modeling integrated the functioning and social behavior factors, grounded in the framework of rehabilitation concepts and the theory of planned behavior. In this research, the decision to utilize SEM over conventional correlation or multiple regression analyses was predicated on its superior capacity to appraise holistic models as opposed to evaluating discrete coefficients [32]. Additionally, SEM’s capability to seamlessly incorporate a multitude of dependent and mediating variables within an integrated analytical framework was a key consideration for our methodological approach [33].

Cronbach’s alpha coefficient was used to evaluate the internal consistency of the scales (reliability). Confirmatory factor analysis was used to test their construct validity. We employed the subsequent statistical model fit indices that have been validated as significant in Structural Equation Modeling (SEM) [34]: the model fit was subsequently appraised using a chi-squared test for goodness-of-fit with a ratio of chi-squared to degrees of freedom < 3, a Goodness-of-Fit Index (GFI) exceeding 0.90, the Root Mean Square Error of Approximation (RMSEA) recommended to be less than 0.05, a Comparative Fit Index (CFI) surpassing 0.90, a Normed Fit Index (NFI) suggested to exceed 0.90, and an Non-Normed Fit Index (NNFI) greater than 0.90. Model fit was confirmed by several statistics summarized in Table 2.

**Table 2:** Standardized item loadings of the final model’s variables

Latent Factors	Items/variants	Standardized factor loading	
		Female	Male
CAOA	Convenience of accessing outdoor activities	0.806	0.791
	Frequency of accessing outdoor activities	0.510	0.605
body pain	Pain magnitude	0.721	0.693
	Pain interference	0.732	0.830
subjective norms	Social respect	0.701	0.796
	Social attitudes	0.867	0.646
positive mental health	Peppy/ Lively	0.556	0.743
	Peaceful	0.739	0.755
	Energetic	0.566	0.577
	Happy	0.703	0.795
IPCPA	intention to participate in community physical activities	0.555	0.537
	Opportunity to participate in community physical activities	0.690	0.479
physical function	Lift, Carry the shopping	0.648	0.846
	Moderate activities	0.794	0.846
	Climb one flight of stairs	0.763	0.839
	Walk one hundred metres	0.838	0.864
	Bathe, dress	0.755	0.766
role emotional	Cut down time	0.912	0.939
	Accomplished less	0.941	0.976
	Not careful	0.955	0.907
negative mental health	Down in the dumps	0.727	0.766
	Blue/ Sad	0.913	0.856
	Worn out	0.655	0.744
medical expenditure	monthly medical spending	0.658	0.540
	maximum annual medical spending	0.468	0.408

The parameter estimation used a maximum likelihood model [31, 35]. In the model, directional arrows illustrate direct causal links where an increase in one variable directly improves another. These relationships could typically exhibit a positive correlation, since we set higher scores indicating enhanced functioning or improved conditions.

## Results

**Fig. 4:**
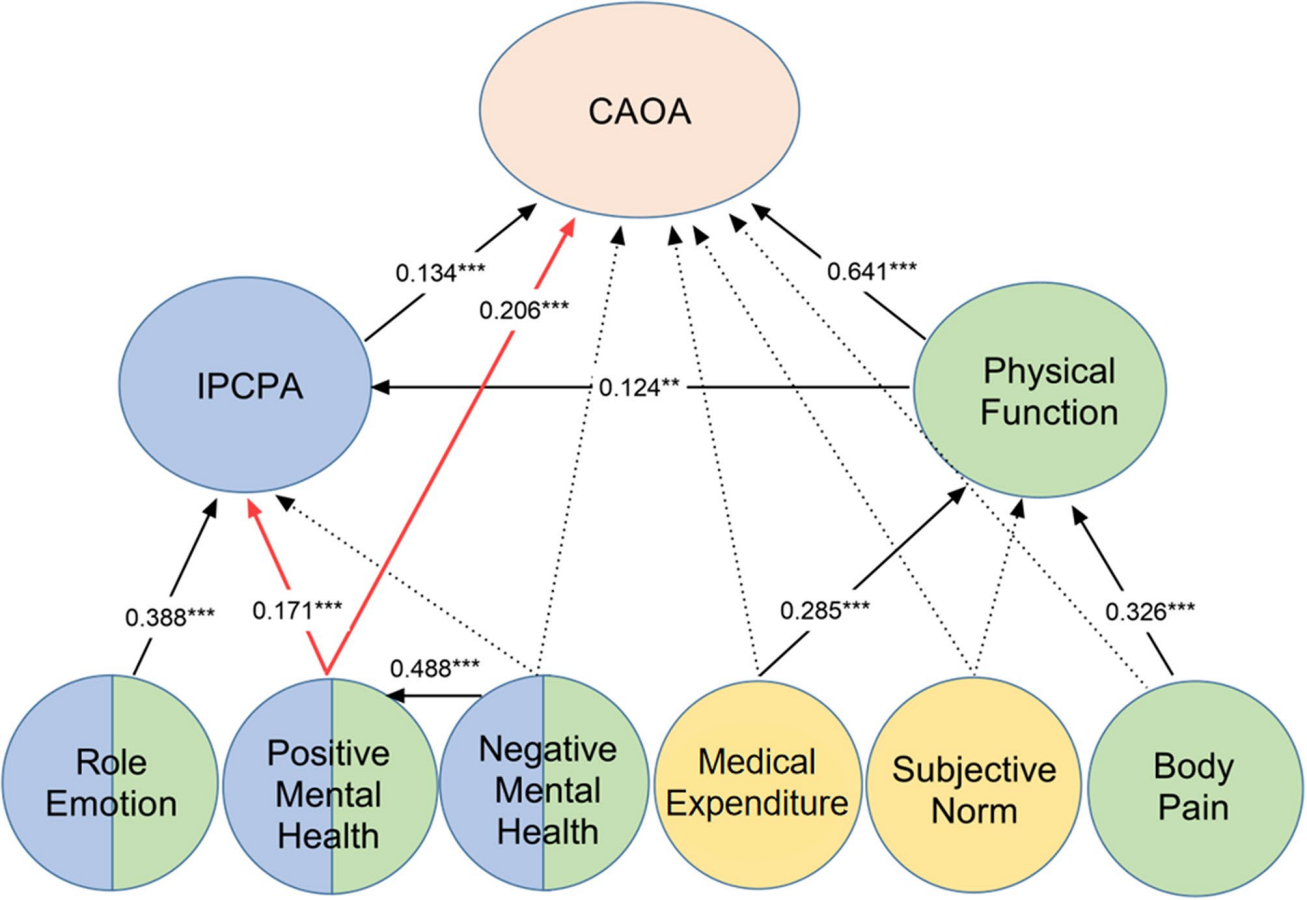
Standardized coefficients relating factors in the final integrated model predicting CAOA for women

Notes. CAOA = convenience of accessing outdoor activities, IPCPA = intention to participate in community physical activities, H = hypothesis.

**Fig. 5:**
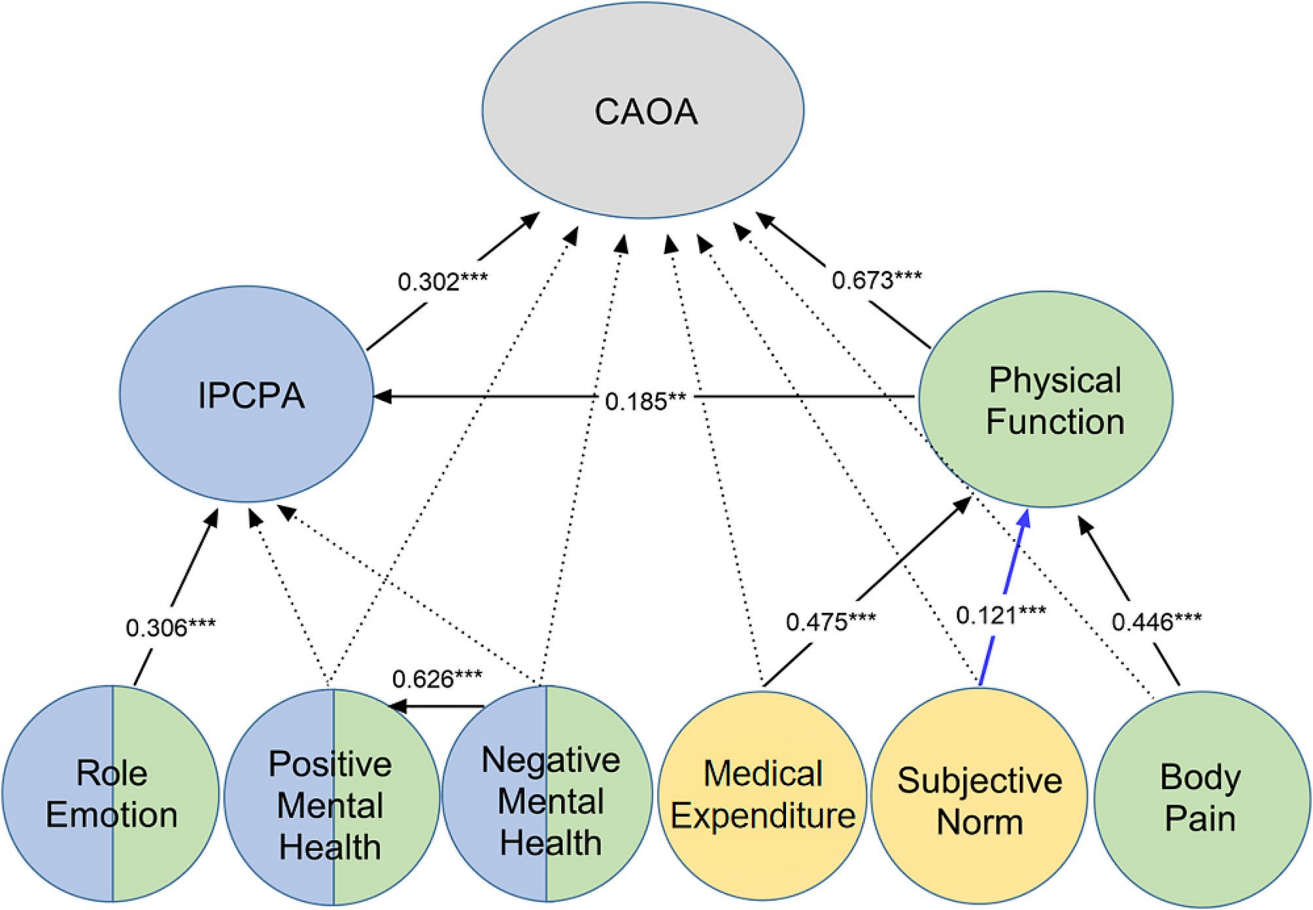
Standardized coefficients relating factors in the final integrated model predicting CAOA for men. *Note* CAOA = convenience of accessing outdoor activities, IPCPA = intention to participate in community physical activities, H = hypothesis ***indicates a relationship significant at the *p* ≤ 0.01 (**p ≤ 0.05) level of confidence. Solid lines indicate significant correlation. Dashed lines indicate no significant correlation and an unsupported hypothesis. Quality of life, social factors and behavioral factors are highlighted in green, yellow and blue respectively. A male-specific significant pathway is shown in blue

Although the mental part of the SF-36 is often divided into mental health and vitality groups, their positive or negative aspects strongly loaded together as one factor based on exploratory factor analysis. The mental variants were therefore grouped into positive mental health and negative mental latent factors instead of mental health and vitality. The re-specified hypothesized model demonstrated good fit. The directions of the relationships among the variables were unchanged, but non-contributing variables were deleted. Therefore, not all original items and domains of the SF-36 were used. Those accepted are shown in Table 3 and highlighted in green in Fig. 1. The final model (shown in Figs. 4 and 5) had nine factors consisting of 25 items with CAOA as the dependent factor. The factors’ Cronbach’s alpha coefficients were all between 0.66 and 0.96, indicating good internal consistency. The standardized factor loadings of the final model ranged from 0.41 to 0.98, indicating that all of the items in the model were valuable predictors of CAOA (Table 3). Both genders showed good model fit.

**Table 3:** Fit indices of the final gender fit modes

	χ^2^/df	GFI	RMSEA	CFI	NFI	NNFI
Female	2.304	0.905	0.048	0.944	0.905	0.932
Male	2.028	0.93	0.043	0.963	0.930	0.955
Fit criteria	< 3	> 0.90	< 0.05	> 0.90	> 0.90	> 0.90

*Note df = degrees of freedom, GFI = goodness-of-fit index, RMSEA = the root mean square approximation error, CFI = comparative fit index, NFI = normed fit index, NNFI = non-normed fit index

Figures 4 and 5 show the following significant relationships supporting the hypotheses. For both women and men around 65% of the variance in CAOA was directly explained by their physical functioning (βwoman = 0.641, βman = 0.673, *p* ≤ 0.01 in both cases), and also by IPCPA (βwoman = 0.134, βman = 0.302, *p* ≤ 0.01 for both). Physical functioning was in turn directly predicted by medical expenditure (βwoman = 0.285, βman = 0.475, *p* ≤ 0.01 for both) and pain (βwoman = 0.326, βman = 0.446, *p* ≤ 0.01 for both) while IPCPA was predicted by the role emotion scores (βwoman = 0.388, βman = 0.306, *p* ≤ 0.01 for both) and by physical functioning (βwoman = 0.124, βman = 0.185, *p* ≤ 0.05 for both). The models also revealed some gender differences, however. In women, positive mental health predicted convenience perceptions (β = 0.206, *p* ≤ 0.01) and intention to participate (β = 0.171, *p* ≤ 0.01); among men, subjective norms correlated significantly with physical functioning (β = 0.121, *p* ≤ 0.05). The female-specific pathways are highlighted in red in Fig. 4, while the male-specific one is highlighted with a blue line in Fig. 5.

Please note that in our study, all factors have been scored such that higher scores indicate enhanced functionality or improved conditions. For instance, the positive correlation between negative and positive mental health observed in both genders indicates that an improvement in negative mental health, when approached healthily, directly contributes to the enhancement of positive mental health.

*** indicates a relationship significant at the *p* ≤ 0.01 (** *p* ≤ 0.05) level of confidence. Solid lines indicate significant correlation. Dashed lines indicate no significant correlation and thus unsupported hypotheses. Quality of life, social factors and behavioral factors are highlighted in green, yellow and blue respectively. Female-specific significant pathways are shown in red.

## Discussion

The gender-specific structural equation models show that SF-36 scores and social-behavioral factors can predict the CAOA for elderly persons with a disability, at least in southern China. The two genders show some common and some different influences.

This study is the first time to have evaluated CAOA among older people with physical disabilities. The findings suggested that although the SF-36 is a valuable and popular instrument, it does not contain enough social context, and it does not aim to predict behavior. In attempting to do so it is important to include other behavioral factors such as IPCPA which influence the CAOA. Social factors such as medical expenditure can also indirectly predict the CAOA via physical functioning. Note that lower medical expenditure cannot be the cause of better physical performance, but there is an undeniable relationship that can be used to predict. The data suggest that a subset of SF-36 scores integrated with social and behavioral factors can usefully predict perceptions of the CAOA and thus behavior. Neither should be neglected in promoting perceptions of convenience among the elderly.

In recent years researchers have shifted from studying the barriers to and facilitators of leisure time physical activities to developing and delivering behavioral interventions [35, 36] such as promoting physical activity for multiple sclerosis based on social cognition theory [37] and for cerebral palsy based on the theory of planned behavior [38]. However, there are still too few studies linking quality of life with social and behavioral factors influencing disabled persons’ physical activity. And a greater range of physical disabilities needs to be studied. This study made a start by integrating quality of life into a social behavior model for various physical disabilities. In doing so it emphasized that whole-person rehabilitation is necessary for improving CAOA among OPWPD.

Generally-recognized environment factors such as bike path width and bus service quality may affect the CAOA. For example Derakhshan’s group reviewed participation facilitators in the context of adapted outdoor physical activity for people with physical disabilities, mainly addressing the social and environmental aspects [5]. But such factors are normally beyond the clinician’s control. This study focused on more controllable factors that might influence rehabilitation strategies. The results are more targeted and helpful for rehabilitation clinicians to improve CAOA among OPWPD.

It is easy to imagine that depression, pain or similar negative mental health conditions could play a significant direct role in influencing perceptions of the CAOA. But the data show that they do not always influence those perceptions directly as physical functioning and IPCPA do. In the analysis of variance in CAOA, physical functioning contributes significantly, with females accounting for 64.1% and males for 67.3% of this variance. On the other hand, the IPCPA also plays a role, contributing to the variance in CAOA, with female counts at 13.4% and male counts at 30.2%. This implies that rehabilitation clinicians must primarily consider not only physical functioning but also behavior intention factors when evaluating and treating elderly with physical disabilities. Our research agrees with a narrative review that highlights the necessity for rehabilitation professionals to deliberately employ strategies aimed at altering behavior, given the challenge of promoting exercise-related behavioral change in rehabilitation settings [39].

That positive mental health was a significant predictor for women (β = 0.488, *p* ≤ 0.01) suggests that focusing on active physical functioning and positive intentions could be helpful in promoting community outdoor rehabilitation activities. If focused entirely on negative mental health risks, organizers of such activities could be missing other important contributing factors such as physical functioning and behavioral intentions, possibly compromising their effectiveness. Pain, understandably, indirectly affects perceptions of convenience by impeding physical functioning, but focusing entirely on limiting pain without otherwise improving physical functioning would probably be sub-optimal.

The group led by Rigby has reviewed the gender disparities in outdoor walking groups, and prior research on gender differences has been largely confined to such case studies. They have shown that male participants often join walking programs to share experiences with other men while women tend to value the social bonds and positive emotions linked to participating in a walking group [21]. The results of current study corroborate observations that male behavior is associated with subjective norms, whereas female behavior is linked to positive emotions. However, this study go beyond by beginning to develop predictive SEM models for gender differences and exploring further the factors involved, each with several variables. The data show that among women, positive mental health affects perceptions of convenience and intentions to participate directly. That seems to fit with the widespread presumption that “women are more emotional than men”. So improving women’s mental health, life satisfaction and happiness should be emphasized in improving female participation in outdoor rehabilitation activities. Among men, subjective norms affect their physical functioning, and then indirectly their perceptions of convenience and their intention to participate. That could be explained by the stereotype of men as needing to appear brave and manly. When subjective norms support a positive attitude towards strength, endurance and stoicism, that encourages men to improve their physical functioning to look more masculine [23]. It is thus important to improve subjective norms about physical functioning to improve the convenience perceptions of elderly persons with a disability.

One limitation of the study is that the subjects were all from a specific rural city in China. Additionally, the sample was restricted to adults with normal intellectual functioning who were able to attend a clinic. Urban settings, by contrast, offer different healthcare resources and social contexts, which could influence the findings. Moreover, individuals with intellectual disabilities may present differently. Caution is thus advised when generalizing these results to other populations. It is also important to bear in mind that the associations observed among SF-36 ratings, IPCPA, and social contexts were cross-sectional. Additional valid longitudinal data are needed. And although the interviewers and data collectors were all especially trained, some self-reporting bias could still have intruded. This study did not include all domains and all variants of the SF-36 in its analyses based on developing the best model fit. The research did not integrate economic and marital status into the SEM analysis, as it was centered on factors of interest to rehabilitation clinicians. Some relationships may therefore have been under-developed. And finally, this study did not evaluate the natural or built environment, which certainly contributes to the objective convenience of accessing outdoor activities. Future studies should consider including this important factor.

## Conclusions

Elderly persons’ perceptions of the convenience of accessing outdoor activities should be given closer attention, particularly in the rehabilitation of those with a disability. An integrated SF-36 and social behavior model based on rehabilitation concepts and planned behavior theory has been shown to usefully predict such perceptions with respect to outdoor activities. The data suggest that physical functioning and IPCPA impact perceptions of the CAOA directly, so they should be the target of interventions. Role emotion, body pain and medical expenditure indirectly influence the perceptions, so they should also be considered in designing outdoor activity interventions. The data show that positive mental health factors can directly affect IPCPA and convenience perceptions among women, while subjective norms are influential among men. This should inspire clinicians to emphasize both health functional status and planned behavior, as well as both gender differences in predicting responses to outdoor rehabilitative activities.

## Data Availability

The data generated in this study are available from the corresponding author TBY on reasonable request.

## Abbreviations

CAOA: convenience of accessing outdoor activities
OPWPD: older people with physical disabilities
SEM: structural equation modeling
H: hypothesis
SF-36: short form 36-item health survey
IPCPA: intention to participate in community physical activities
